# Knockout of *bcas3* gene causes neurodevelopment defects in zebrafish

**DOI:** 10.1186/s40659-025-00615-4

**Published:** 2025-06-06

**Authors:** Huihui Liu, Nianyi Sun, Zhenxing Liu, Jinze Li, Xianqin Zhang

**Affiliations:** Key Laboratory of Molecular Biophysics of the Ministry of Education, College of Life Science and Technology, Center for Human Genome Research, Huazhong University of Science and Technology, Wuhan, 430074 China

**Keywords:** BCAS3, CRISPR/Cas9, Zebrafish, Neurodevelopmental disorder

## Abstract

**Background:**

Neurodevelopmental disorders manifest in early childhood and are characterized by cognitive deficits, intellectual disabilities, motor disorders, and social dysfunction. Mutations in *BCAS3* gene are associated with syndromic neurodevelopmental disorders in humans, while the detailed pathological mechanism is still unknown.

**Methods:**

CRISPR/Cas9 technology was used to generate a *bcas3* knockout zebrafish model. To investigate the effects of *bcas3* on development, morphological evaluations were conducted. Locomotor behaviors, including performance in the light-dark test, novel tank test, mirror test, shoaling test, and social test, were assessed through video tracing and quantitative analysis of movement parameters. Transcriptome sequencing analysis was used to identify dysregulated pathways associated with development process. Additionally, Acridine Orange staining was employed to evaluate apoptosis. Western blot and real-time RT-PCR were used to analyze the expression levels of genes.

**Results:**

*Bcas3* knockout zebrafish exhibited early larval phenotypes resembling clinical features of patients with *BCAS3* mutations, including global delayed development at early embryonic development, microcephaly and reduced body length. Behavior analysis revealed abnormal motor dysfunction, such as social impairment, increased anxiety and heightened aggression. Notably, human *BCAS3* rescued the developmental defects and motor disorders in *bcas3* knockout larvae. Transcriptomic analysis identified substantial downregulation of genes related to embryonic development and startle response, brain development and neuron migration in *bcas3* knockout zebrafish, such as *rpl10*, *cyfip2*, *erbb3b*, *eya4a*, *nr2f1b*, *prkg1b* and *ackr3b*. Additionally, increased apoptosis was observed in *bcas3* knockout zebrafish, which was further confirmed by Acridine Orange staining and a decreased Bcl2/Bax ratio in western blot analysis. The increased apoptosis observed in the brain of *bcas3* knockout larvae could contribute to the developmental and locomotor deficits.

**Conclusion:**

The *bcas3* knockout zebrafish model recapitulates the clinical features observed in patients with *BCAS3* mutations. Our results suggest that increased apoptosis may underlie the developmental deficits and motor disorders in these patients. The *bcas3* knockout zebrafish model provides a valuable tool to identify dysregulated molecular targets for therapeutic intervention during the early stages of disease progression.

**Supplementary Information:**

The online version contains supplementary material available at 10.1186/s40659-025-00615-4.

## Introduction

Neurodevelopmental disorders (NDDs) are complex conditions that usually occur in childhood and involve disorders of brain development affecting cognitive and motor functions [1, 2]. NDDs include a variety of diseases, such as intellectual disability, autism, epilepsy and other rare genetic developmental disorders [2–4]. The symptoms and functional impairment of neurodevelopmental disorders typically persist into adolescence and adult life, imposing heavy burden to patients, families and society [5, 6].

The pathogenesis of neurodevelopmental disorders is extremely complex, involving multiple factors such as genes, environment and immunity [7–10]. Mutations in *BCAS3* (BCAS3 microtubule associated cell migration factor) lead to neurodevelopmental disorders [11–14]. The patients with *BCAS3* mutations presented with global growth retardation accompanied by microcephaly, short stature and seizures [12, 14, 15]. BCAS3 protein participates in cell migration and angiogenesis by interacting with cytoskeletal components to maintain microtubule dynamics [16–18]. *Bcas3* deficiency in mice caused embryonic lethality due to abnormal angiogenesis [19]. Comprehending the function of *BCAS3* is crucial in uncovering its involvement in diverse biological processes.

In this study, we constructed a *bcas3* knockout (KO) zebrafish model to investigate the potential mechanism of *BCAS3* deficiency by which causes neurodevelopmental disorders. Our results revealed that *bcas3* deficits in zebrafish led to neurodevelopmental deficiency and abnormal behavior phenotypes. Notably, human *BCAS3* could rescue both the microcephaly and behavior abnormalities in *bcas3* KO zebrafish. RNA sequencing analysis revealed that *bcas3* KO disrupted the expression of genes associated with startle response, embryonic brain development and neuron migration in zebrafish, accompanied by significantly increased apoptosis in the brain of *bcas3* KO zebrafish. These findings highlight the critical role of BCAS3 in brain development.

## Results

### Generation of *bcas3* KO zebrafish using CRISPR/Cas9

**Fig. 1:**
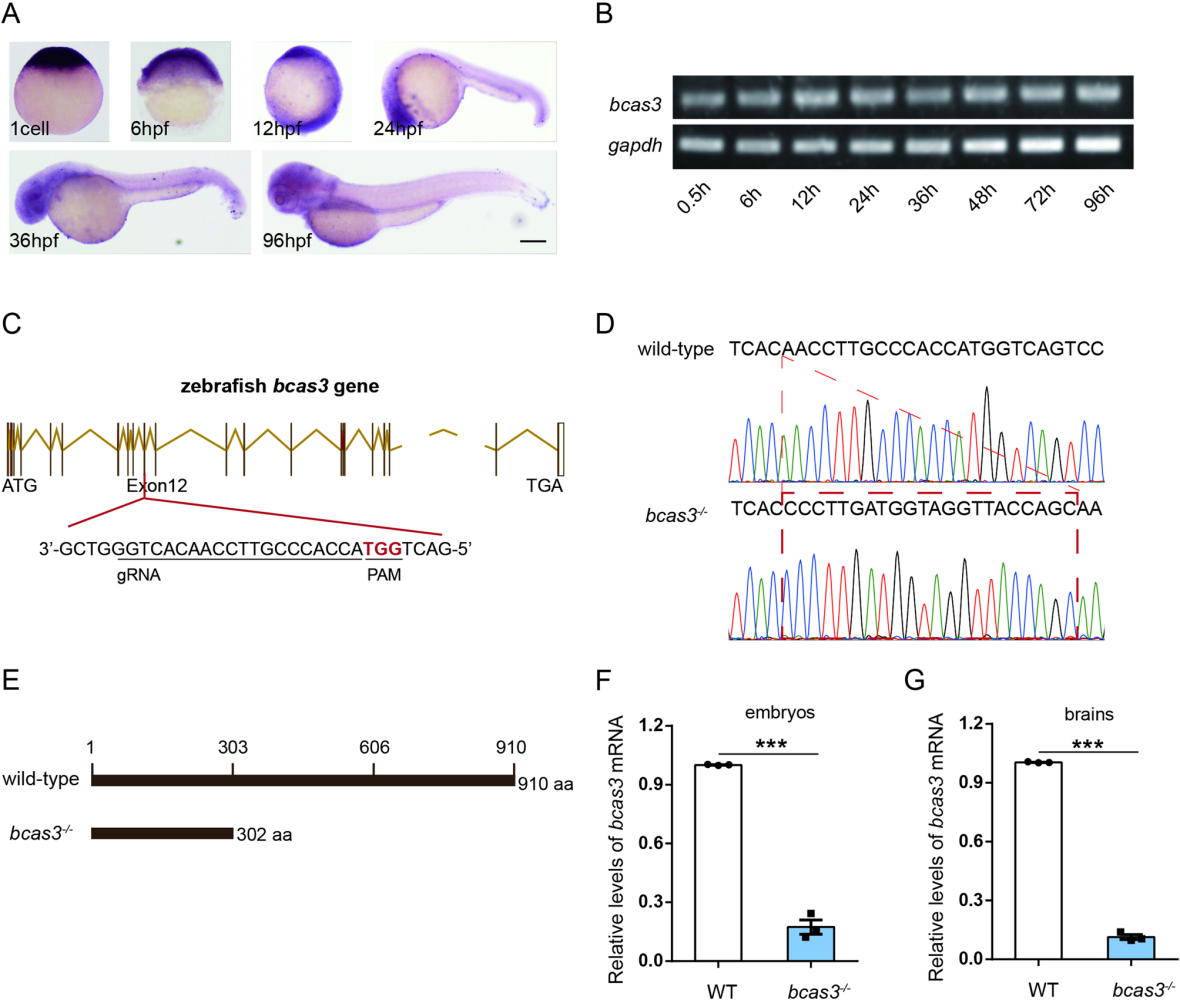
Construction of *bcas3* KO zebrafish by CRISPR/Cas9 technology. **(A)**, Whole-mount in situ hybridization (WISH) for *bcas3* mRNA in WT zebrafish embryos at different development stages. Scale bars: 200 μm. (*n* = 20 embryos per group). WT: wild type. **(B)**, Temporal pattern of mRNA expression of zebrafish *bcas3* gene at different stages. mRNA extracts from whole-embryos were analyzed by RT-PCR for *bcas3* mRNA levels at different zebrafish embryo stages. (*n* = 20 embryos per group). **(C)**, Schematic diagram shows the genomic structure of zebrafish *bcas3* with all 24 exons and the site of genome editing marked. The gene editing target site is located in the 12th exon of *bcas3* gene. Target sequences are underlined. PAM sequences are highlighted in red. **(D)**, DNA sequence analysis identified the *bcas3*^*−/−*^ insertion mutant zebrafish line with a 22 bp insertion in exon 12 of *bcas3* gene. **(E)**, The 22 bp insertion in *bcas3* causes frame-shift and is predicted to cause a premature stop codon, resulting in a truncated mutant bcas3 proteins with only 302 amino acids left. **(F-G)**, Real-time RT-PCR analysis showed that the *bcas3* mRNA level in 10 hpf (hours post fertilization) embryos and brains of 4 mpf (months post fertilization) *bcas3*^*−/−*^ zebrafish was reduced compared to WT controls. (*n* = 3, per group). The *rpl13a* gene was used as endogenous control. Data are shown as mean ± SEM. Unpaired Student’s t-test was used to analyze RT-qPCR. Significance levels are denoted as follows: **P < 0.01, ***P < 0.001

Zebrafish Bcas3 protein shares 85.16% amino acid sequence identity with human BCAS3 protein. To clarify the role of zebrafish *bcas3* gene, the spatial and temporal expression patterns during embryonic development were examined by whole-mount in situ hybridization (WISH) and RT-PCR. Expression of *bcas3* was detected at one-cell stage. From 0.5 hpf (hour post fertilization) to 12 hpf, *bcas3* expression was widely distributed in embryos. From 24 hpf to 72 hpf, *bcas3* was expressed predominantly in the brain (Fig. 1A). RT-PCR showed that *bcas3* was expressed throughout embryonic development (Fig. 1B).

To investigate the mechanism by which *bcas3* deficiency leads to neurodevelopmental disorders, *bcas3* KO zebrafish was generated using CRISPR/Cas9 technology (Fig. 1C). The *bcas3*^*−/−*^ strain harbored a 22 bp insertion in exon 12, resulting in a premature stop codon and a truncated protein with 302 amino acids (Fig. 1D-E). Real-time RT-PCR analysis showed that the expression level of *bcas3* mRNA was dramatically reduced in *bcas3*^*−/ −*^zebrafish, suggesting that the 22 bp insertion causes nonsense-mediated decay of *bcas3* mRNA (Fig. 1F-G).

### *Bcas3* deficiency leads to developmental abnormalities in zebrafish

**Fig. 2:**
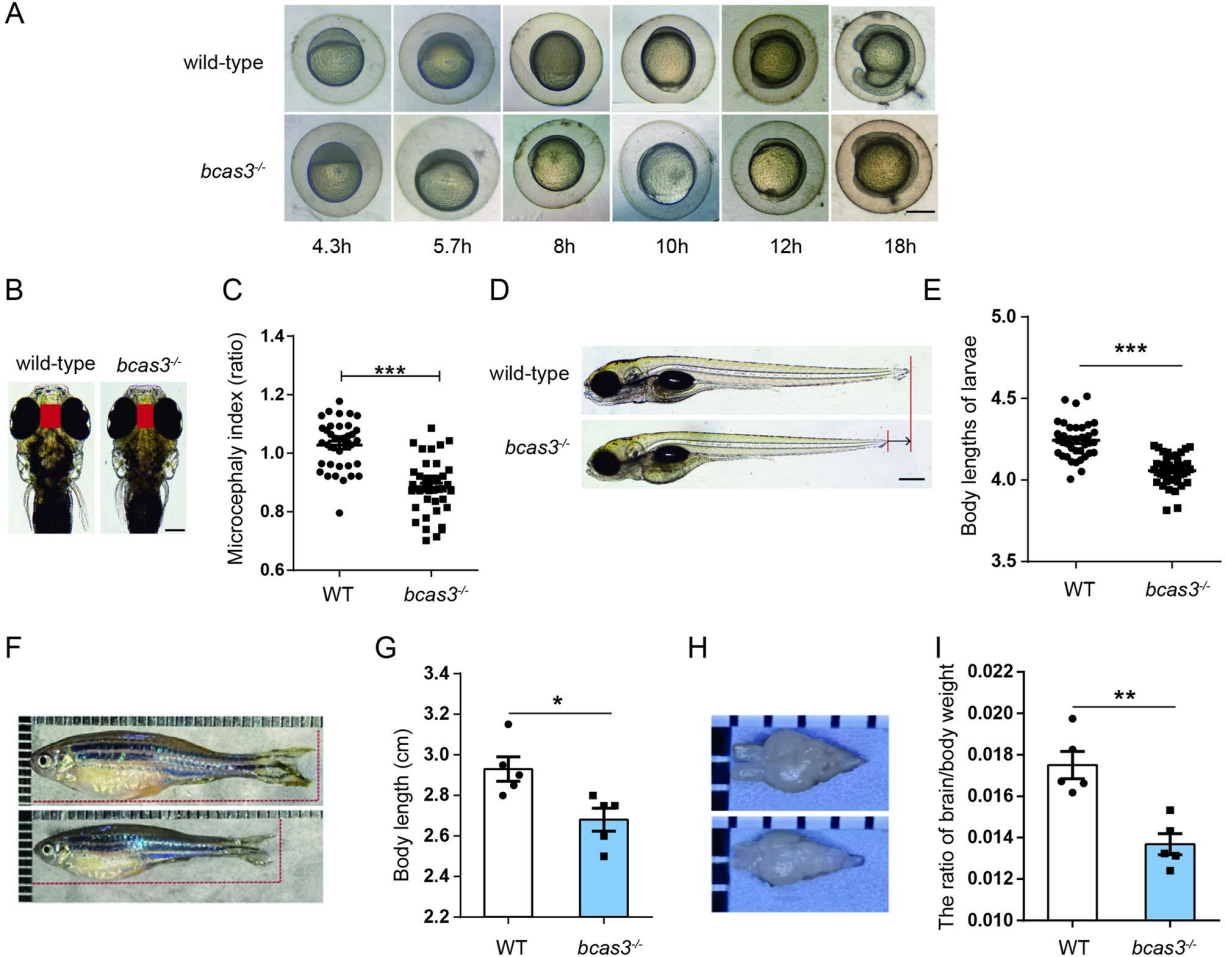
*Bcas3* KO in zebrafish results in developmental deficiency. **(A)**, Time-matched bright field images of WT and *bcas3*^*−/−*^ embryos during early development. Scale bars: 200 μm. (*n* = 15, per group). (**B-C)**, Microcephaly index (the interocular distances/body length) comparison of 5 dpf (days post fertilization) WT and *bcas3*^*−/−*^ larvae. Representative images **(B)** and quantitative analysis **(C)** of the interocular distances/body length in 5 dpf WT (*n* = 37) and *bcas3*^*−/−*^ larvae (*n* = 41). Scale bars: 500 μm. **(D-E)**, Body length comparison of 5 dpf WT and *bcas3*^*−/−*^ larvae. Representative images **(D)** and quantitative analysis of body lengths **(E)** in 5 dpf WT (*n* = 37) and *bcas3*^*−/−*^ larvae (*n* = 41). Scale bars: 200 μm. **(F-G)**, Body length comparison of 4 mpf WT and *bcas3*^*−/−*^ larvae. Representative images **(F)** and quantitative analysis of body lengths **(G)** in 4 mpf zebrafish. Scale bars: 200 μm. (*n* = 5, per group). (**H)**, Representative images of the brain of 4 mpf zebrafish. **(I)**, The ratio of brain/body weight of WT and *bcas3*^*−/−*^ zebrafish at 4 months. (*n* = 5, per group). Data are shown as mean ± SEM. Unpaired Student’s t-test was used to analyze interocular distances, body length, and the ratio of brain/body weight. Significance levels are denoted as follows: *P < 0.05, ***P < 0.001

Compared to wild-type, maternal *bcas3*^*−/−*^ embryos showed delayed early-stage development (Fig. 2A). The *bcas3*^*−/−*^ embryos developed at the same rate as wild-type embryos until the sphere stage at 4.3 hpf, but exhibited a slight delay afterward (Fig. 2A). In particular, when wild-type embryos reached the 75%-epiboly stage at 8 hpf, the *bcas3*^*−/−*^ embryos remained at the shield stage at 6 hpf (Fig. 2A). Subsequently, the *bcas3*^*−/−*^ embryos consistently showed a significant delay equivalent to 1.5–2 h of normal development compared to the wild-type embryos (Fig. 2A). Microcephaly phenotype, characterized as a microcephaly index (interorbital distance/ full body length) [20], was detected in 5 dpf (days post fertilization) *bcas3*^*−/−*^ larvae (Fig. 2B-C). During the larval stage, 5 dpf *bcas3*^*−/−*^ larvae displayed significantly shorter body lengths than wild-type larvae (Fig. 2D-E). In the adult stage, the body length of 4 mpf (months post fertilization) *bcas3*^*−/−*^ zebrafish was shorter than that of wild-type adults (Fig. 2F-G) and the brain weight of *bcas3*^*−/−*^ adults was significantly decreased (Fig. 2H-I).

### *Bcas3* KO zebrafish larvae showed locomotor hyperactivity

**Fig. 3:**
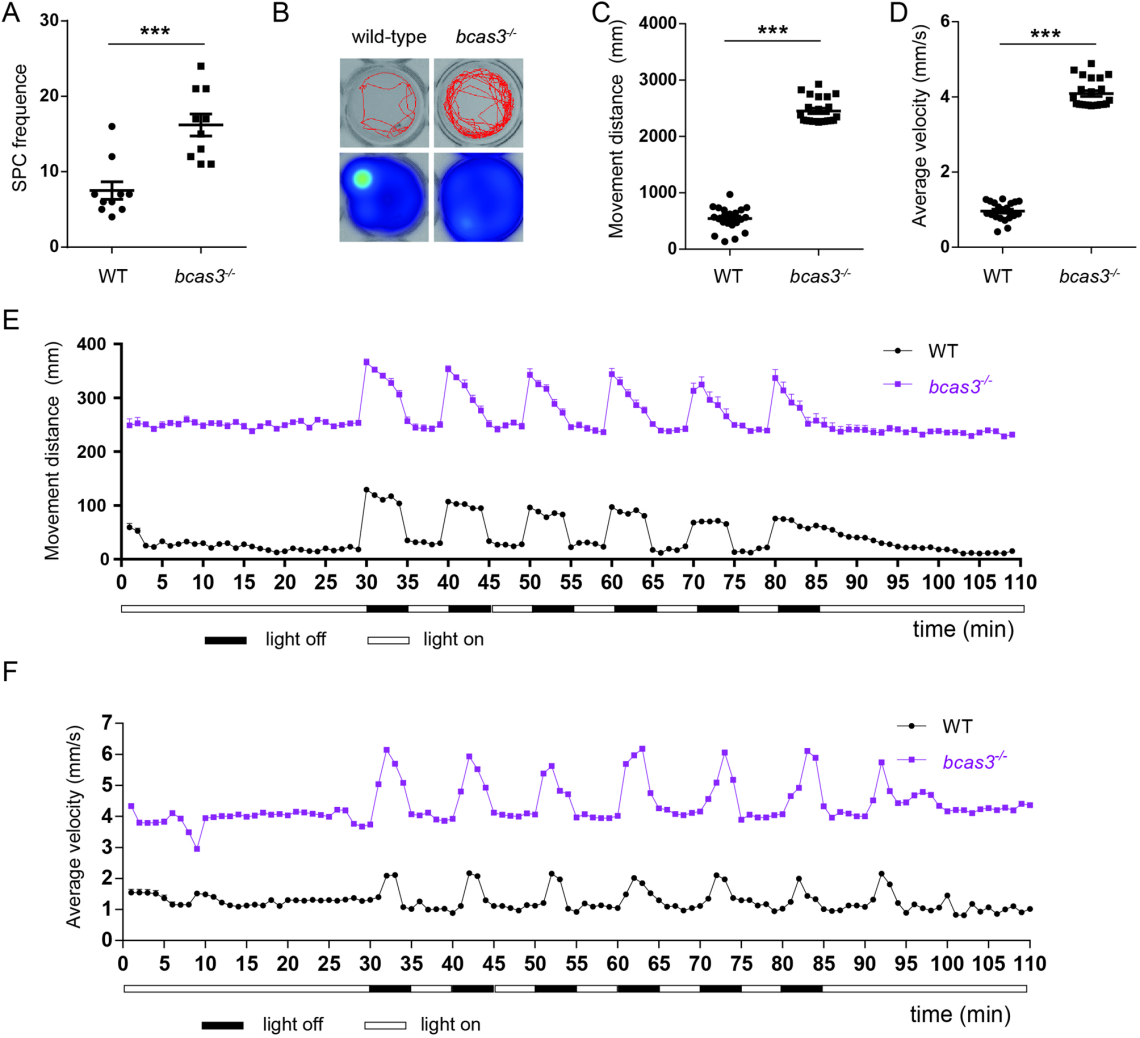
The locomotor activity of *bcas3* KO zebrafish larvae. **(A)**, The SPC frequence of 24 hpf zebrafish. SPC, spontaneous curls. (*n* = 10, per group). **(B-D)**, The basic movement of zebrafish under light condition. **(B)**, Representative image of trajectory and heat map. Quantitative analysis of distance **(C)** and average velocity **(D)** in 6 dpf WT and *bcas3*^*−/−*^ larvae. (*n* = 22, per group). **(E)**, Graph illustrating the movement distance of 6 dpf zebrafish to alternating light-dark conditions, with periods consisting of 5-minute darkness and 5-minute light. The plot graph represents the movement distance of WT and *bcas3*^*−/−*^ larvae per minute. (*n* = 23, per group). (**F)**, The average velocity of 6 dpf zebrafish during light-dark conditions with periods consisting of 55-minute darkness and 5-minute light. The plot graph represents the average velocity of WT and *bcas3*^*−/−*^ larvae per minute. (*n* = 23, per group). Data are shown as mean ± SEM. Unpaired Student’s t-test was used to analyze the SPC frequence, distance and average velocity. Significance levels are denoted as follows: ***P < 0.001

The spontaneous curls is an important reflection response in fish embryos to study neurodevelopmental behavior [21, 22]. The frequency of tail swing of 24 hpf *bcas3*^*−/−*^ larvae was higher than that of wild-type larvae (Fig. 3A). The locomotor behavior of zebrafish larvae reflects the health and physiological state [23]. Increased locomotor activity was displayed in *bcas3*^*−/−*^ larvae during test (Fig. 3B). Additionally, both movement distance and velocity of 6 dpf *bcas3*^*−/−*^ larvae were significantly increased (Fig. 3C-D). Larval locomotor ability and resilience to stressful stimuli were monitored in response to light-dark transitions. Both wild-type and *bcas3*^*−/−*^ larvae showed increased movement from light-to-dark transitions and decreased movement from dark-to-light transitions (Fig. 3E). However, the *bcas3*^*−/−*^ larvae showed a significant increase in movement distance during light-dark transitions compared to wild-type larvae (Fig. 3E). Additionally, the average velocity of *bcas3*^*−/−*^ larvae increased during the light-dark test (Fig. 3F). These results suggested that *bcas3* deficiency resulted in alterations in locomotor behavior.

### *Bcas3* KO increases anxiety-like and aggressive behavior in zebrafish

**Fig. 4:**
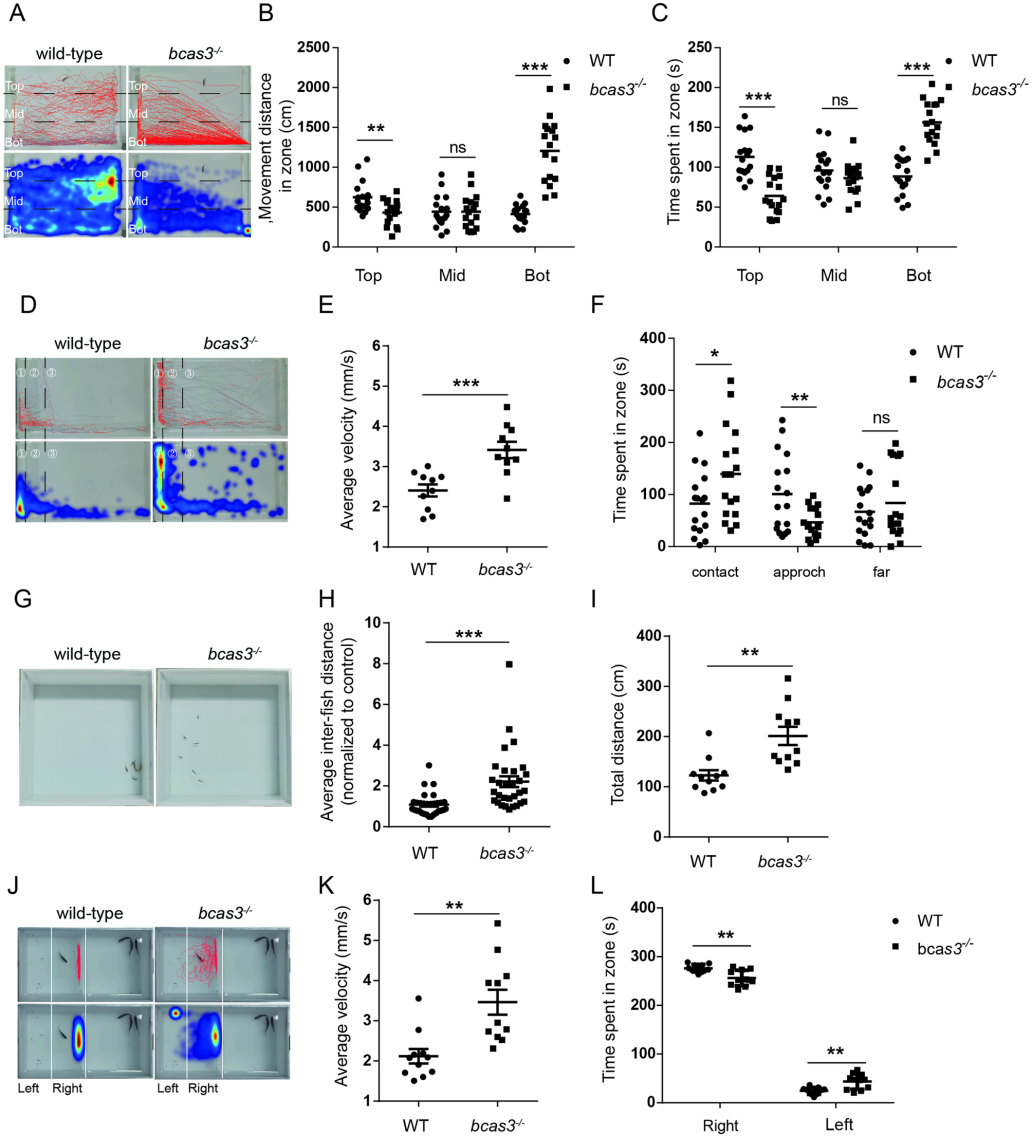
Adult *bcas3* KO zebrafish showed increased anxiety, aggression and abnormal social. **(A-C)**, Analysis of the behavior of 4 mpf WT and *bcas3*^*−/−*^ zebrafish in novel tank test. **(A)**, Representative trajectory and heatmap image. Quantitative analysis of distance **(B)** and time **(C)** spent in bottom, middle, and top zones. (*n* = 17, per group). *Bcas3* KO zebrafish exhibited reduced distance and movement time spent in the top zone compared to WT controls, indicating increased anxiety-like behavior. The tank was divided horizontally into three equal sections: the top zone, the middle zone, and the bottom zone. (**D-F)**, Analysis of 4 mpf WT and *bcas3*^*−/−*^ zebrafish behavior in mirror biting test. **(D)**, Representative trajectory and heatmap image. Quantitative analysis of average velocity **(E)** and active time spent in contact zone (the distance from the mirror < 2 cm), approach zone (2 cm ≤ the distance from the mirror ≤ 6 cm), and far zone (the distance from the mirror > 6 cm) **(F)** in WT and *bcas3*^*−/−*^ zebrafish. ①, contact. ②, approach. ③, far. (*n* = 17 per group). *Bcas3* KO zebrafish spent more time in the contact zone, indicating heightened aggressive behavior. (**G-H)**, Analysis of 4 mpf WT and *bcas3*^*−/−*^ zebrafish behavior in shoaling behavior test. Representative image **(G)** and average inter-fish distance **(H)** was significantly increased in *bcas3*^*−/−*^ zebrafish, indicating reduced social cohesion. (*n* = 30, per group). **(I-L)**, Analysis of 4 mpf WT and *bcas3*^*−/−*^ zebrafish behavior in social test. **(J)**, Representative trajectory and heatmap image. Quantitative analysis of total movement distance **(I)**, average velocity **(K)** and time spent in left and right regions **(L)** in WT and *bcas3*^*−/−*^ zebrafish. (*n* = 11, per group). Data are shown as mean ± SEM. Unpaired Student’s t-test was used to analyze movement distance, average velocity and active time spent in different zones. Significance levels are denoted as follows: *P < 0.05, **P < 0.01, ***P < 0.001. ns, not significant

After the larval locomotor tests, adult zebrafish behavior tests were conducted to assess the impact of *bcas3* gene deficiency on the behavior. The novel tank test was carried out to evaluate anxiety levels by assessing innate adaptive ability in a new environment [24]. Reduced exploratory behavior was displayed in *bcas3*^*−/−*^ zebrafish during the test (Fig. 4A). *Bcas3*^*−/−*^ zebrafish traveled shorter distances (Fig. 4B) and spent less time in the top of the tank (Fig. 4C), indicating reduced exploratory behavior and increased anxiety-like behavior.

The mirror test is an assay used to measure aggressive behavior and it is known that the test zebrafish recognize the opponent in the mirror as an unfamiliar zebrafish [25]. As a result, the movement behavior of *bcas3*^*−/−*^ zebrafish was drastically different from that of wild-type zebrafish. Both wild-type and *bcas3*^*−/−*^ zebrafish showed an interaction with the mirror (Fig. 4D). During the test period, the average velocity of *bcas3*^*−/−*^ zebrafish was increased compared to that of wild-type zebrafish (Fig. 4E). Additionally, *bcas3*^*−/−*^ zebrafish exhibited increased time spent in the contact zone and a mildly decreased in time spent in the approach zone (Fig. 4F). These results suggested increased anxiety and aggression in *bcas3*^*−/−*^ zebrafish.

### *Bcas3* KO zebrafish displayed decreased social interactions

Zebrafish are highly social animals, shoaling tests and social interaction tests were conducted to observe social behavior [26]. The shoaling test showed that *bcas3*^*−/−*^ zebrafish formed a loose shoal, as indicated by a higher average inter-fish distance (Fig. 4G-H). Increased locomotor activity was displayed by *bcas3*^*−/−*^ zebrafish (Fig. 4J), supported by a higher movement distance (Fig. 4I) and average velocity compared to wild-type zebrafish (Fig. 4K). Additionally, the time spent approach the separator by *bcas3*^*−/−*^ zebrafish was reduced compared to wild-type zebrafish (Fig. 4L). These results suggested that *bcas3* deficiency causes reduced social interactions.

### Human *BCAS3* rescued the developmental abnormalities and locomotor defects in *bcas3* KO larvae

**Fig. 5:**
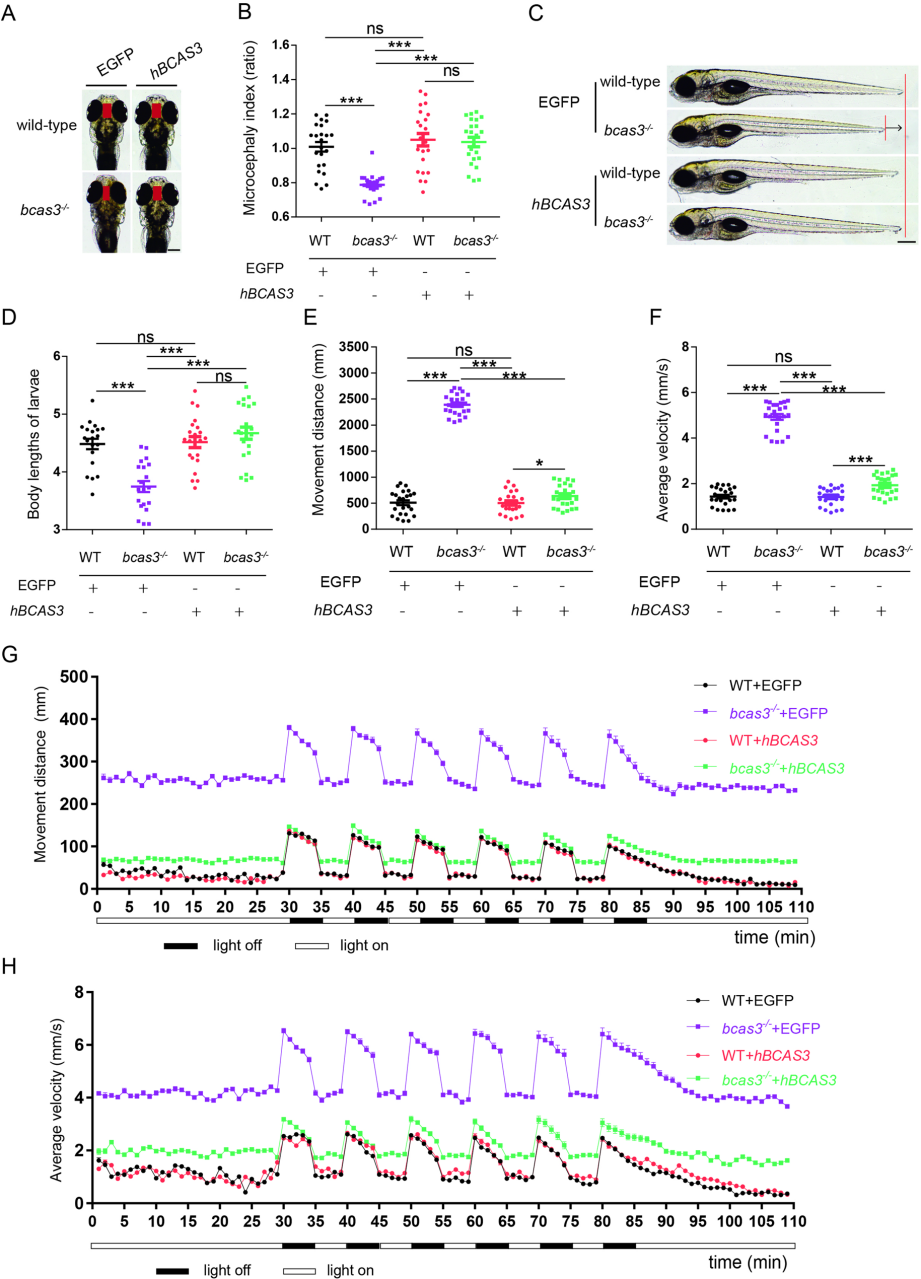
Human *BCAS3* gene rescued the developmental deficiency and locomotor defects of *bcas3* KO larvae. (**A-B)**, Microcephaly index (the interocular distances/body length) comparison of 5 dpf WT and *bcas3*^*−/−*^ larvae injected with EGFP or EGFP*-hBCAS3*. Representative images **(A)** and quantitative analysis of the interocular distances/body length in 5 dpf larvae, (*n* = 23, per group). *hBCAS3*, human BCAS3. (**C-D)**, Body length comparison of 5 dpf WT and *bcas3*^*−/−*^ larvae injected with EGFP or *hBCAS3*. Representative images **(C)** and quantitative analysis of body lengths **(D)** in 5 dpf larvae. WT + EGFP (*n* = 20), *bcas3*^*−/−*^ + EGFP (*n* = 20), WT + EGFP*-hBCAS3* (*n* = 22), *bcas3*^*−/−*^ + EGFP*-hBCAS3* (*n* = 22). Scale bars: 500 μm. **(E-F)**, The basic movement of zebrafish, injected with EGFP or EGFP*-hBCAS3*, under light condition. Quantitative analysis of distance **(E)** and average velocity **(F)** in 6 dpf WT and *bcas3*^*−/−*^ larvae. (*n* = 24, per group). **(G)**, Graph illustrating the movement distance of 6 dpf zebrafish, injected with EGFP or EGFP*-hBCAS3*, to alternating light-dark conditions, with periods consisting of 5-minute darkness and 5-minute light. The plot graph represents the movement distance of larvae per minute. WT + EGFP (*n* = 24), *bcas3*^*−/−*^ + EGFP (*n* = 24), WT + EGFP*-hBCAS3* (*n* = 23), *bcas3*^*−/−*^ + EGFP*-hBCAS3* (*n* = 22). (**H)**, Graph illustrating the average velocity of 6 dpf zebrafish, injected with EGFP or EGFP*-hBCAS3*, to alternating light-dark conditions, with periods consisting of 5-minute darkness and 5-minute light. The plot graph represents the average velocity of larvae per minute. WT + EGFP (*n* = 24), *bcas3*^*−/−*^ + EGFP (*n* = 24), WT + EGFP*-hBCAS3* (*n* = 23), *bcas3*^*−/−*^ + EGFP*-hBCAS3* (*n* = 22). Data are shown as mean ± SEM. One-way ANOVA was used to analyze interocular distances, body length, movement distance and average velocity. Significance levels are denoted as follows: ***P < 0.001. ns, not significant

To further demonstrate that the decreased *bcas3* expression accounts for the developmental abnormalities and locomotor defects in *bcas3* KO larvae, we constructed human *BCAS3* gene overexpression plasmid (pTol2-BCAS3-T2A-EGFP). Human *BCAS3* was injected into one-cell stage zebrafish embryos. At 24 hpf, the embryos with GFP green fluorescence were selected to assess the morphology and locomotor behavior. The *bcas3*^*−/−*^ embryos with overexpression of human *BCAS3* showed increased interorbital distance and body lengths as compared to those in the GFP group (Fig. 5A-D). Additionally, 6 dpf *bcas3*^*−/−*^ larvae with overexpression of human *BCAS3* exhibited significantly reduced movement distance and velocity compared to that with GFP group (Fig. 5C-D). Similarly, overexpression of human *BCAS3* in *bcas3*^*−/−*^ larvae significantly reduced the movement distance and average velocity in dark conditions during light-dark transitions compared to those in the GFP group (Fig. 5G-H). These data suggest that overexpression of human *BCAS3* can rescue the developmental abnormalities and locomotor defects phenotype in *bcas3* KO zebrafish.

### *Bcas3* KO predominantly downregulates genes related with neurodevelopment in zebrafish

**Fig. 6:**
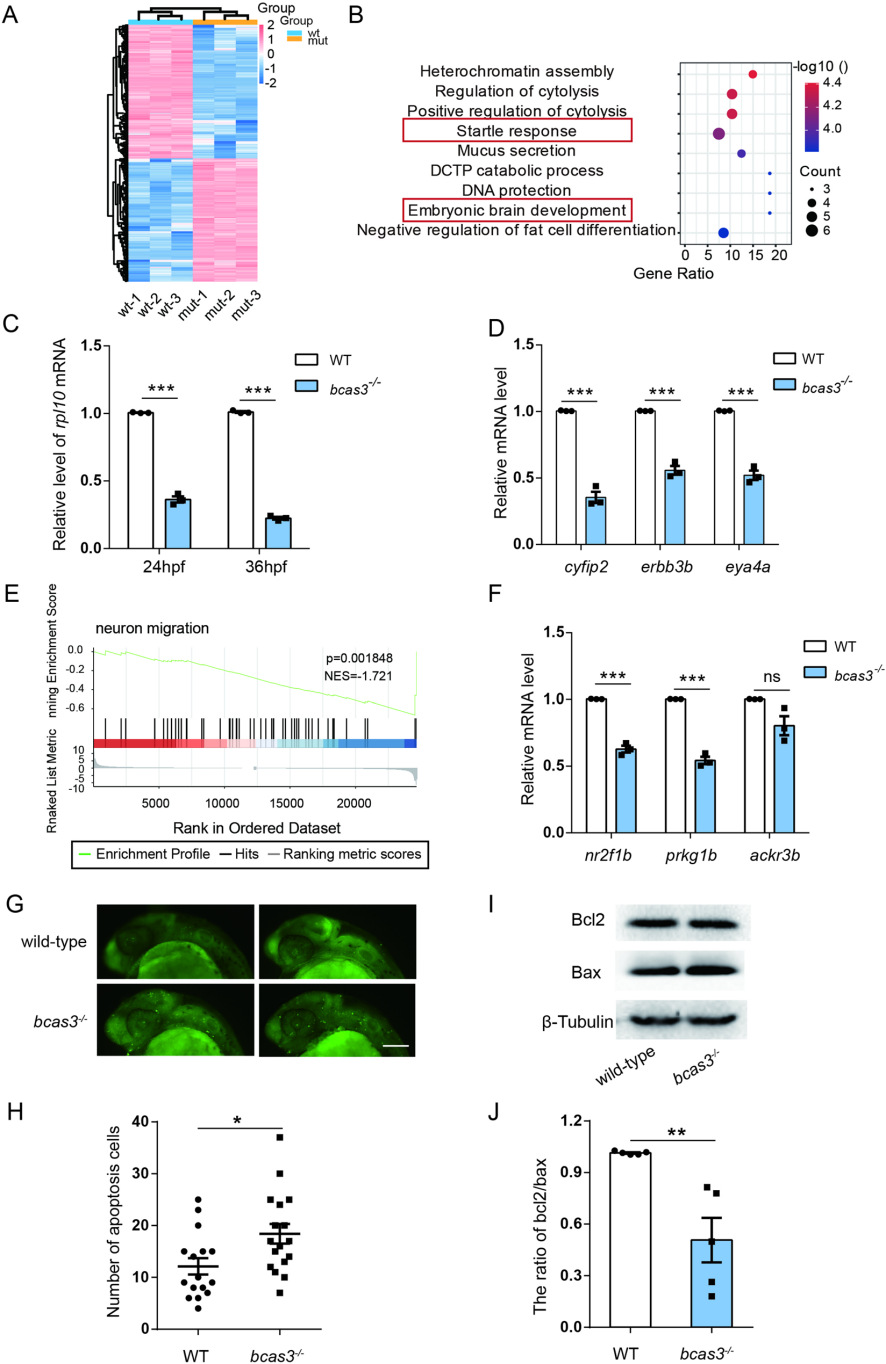
Transcriptomic profiling analysis of *bcas3* KO zebrafish larvae. **(A)**, Hierarchical clustering heat map of 920 upregulated and 998 downregulated genes from 3dpf *bcas3*^*−/−*^ larvae comparison to WT controls (*n* = 30, per group). Color intensity indicating fold changes. Upregulation and downregulation are marked in *pink* and *blue*, respectively. WT: wild-type larvae; mut: *bcas3*^*−/−*^ larvae. **(B)**, ClueGO pathway analysis. **(C)**, Real-time RT-PCR analysis showed that the *rpl10* mRNAlevel in 24 hpf embryos and 36 hpf embryos from *bcas3*^*−/−*^ zebrafish was reduced compared to WT controls. (*n* = 3, per group). The *rpl13a* gene was used as endogenous control. **(D)**, Real-time RT-PCR analysis for *cyfip2*, *erbb3b* and *eya4* mRNA in 36 hpf zebrafish. (*n* = 3, per group). The *rpl13a* gene was used as endogenous control. **(E)**, GESA analysis revealed that *bcas3* KO affected expression of genes involved in neuron migration. GESA, gene set enrichment analysis. **(F)**, Real-time RT-PCR analysis for *nr2f1b*, *prkg1b* and *ackr3b* mRNA in 3 dpf zebrafish. (*n* = 3, per group). The *rpl13a* gene was used as endogenous control. **(G-H)**, 36 hpf WT and *bcas3*^*−/−*^ larvae were stained with acridine orange (AO) to label apoptosis cells in vivo. Representative images **(G)** of AO staining in the larvae brains as indicated. Scale bars: 200 μm. **(H)**, Quantitative analysis of apoptotic cells in the brain areas of the WT (*n* = 16) and *bcas3*^*−/−*^ larvae (*n* = 17). **(I-J)**, Western-blot analysis for apoptosis- relative protein Bcl2 and Bax in 3 dpf zebrafish. (*n* = 5, per group). β-Tubulin was used as endogenous control. Data are shown as mean ± SEM. Unpaired Student’s t-test was used to analyze RT-qPCR, AO staining, and western blot. Significance levels are denoted as follows: *P < 0.05, **P < 0.01, ***P < 0.001. ns, not significant

To investigate the molecular basis of neurodevelopmental deficits caused by *bcas3* KO, we conducted transcriptome analysis by RNA-seq on 3 dpf wild-type and *bcas3*^*−/−*^ larvae. RNA sequencing data revealed 1918 differentially expressed genes in *bcas3*^*−/−*^ larvae compared to wild-type larvae, with |log2 fold changes | ≥ 2 and P values ≤ 0.01, including 920 upregulated and 998 downregulated genes (Fig. 6A).

GO (Gene Ontology) analysis of these differentially expressed genes identified top-ranking clusters predominantly associated with startle response and embryonic brain development (Fig. 6B). RNA-seq and real-time RT-PCR analysis confirmed the downregulation of key genes involved in startle response and embryonic brain development genes, including *cyfip2*, *erbb3b*, *eya4* and *rpl10* (Fig. 6C-D). These findings demonstrate that *bcas3* KO results in a substantial downregulation of genes essential for brain development. Interestingly, GSEA (Gene Set Enrichment Analysis) showed that *bcas3* KO affects the expression of genes related to neuron migration (Fig. 6E). Real-time RT-PCR analysis further confirmed that *nr2f1b*, *prkg1b* and *ackr3b* which are associated with cell migration and proliferation were significantly downregulated in *bcas3*^*−/−*^ larvae (Fig. 6F).

BCAS3 inhibits apoptosis by promoting p53 ubiquitination through CRL4A complex [27–29]. And, deficiency of *RPL10* has been shown to induce apoptosis in zebrafish brain [30]. To investigate apoptosis, wild-type and *bcas3*^*−/−*^ larvae at 36 hpf were stained with Acridine Orange (AO) fluorescent dye. The apoptotic spots in the brains of *bcas3*^*−/−*^ larvae were clearly visible and significantly increased compared to those in wild-type larvae (Fig. 6G-H). Western blot analysis showed that the expression of Bax in 3 dpf *bcas3*^*−/−*^ larvae was significantly increased, and the ratio of Bcl2/Bax was decreased compared to wild-type larvae (Fig. 6I-J). Taken together, these data suggested that *bcas3* KO increased the cell apoptosis in brain leading to neurodevelopmental deficiencies.

## Discussion

Although some functional roles of BCAS3 have been implied in mouse studies, no adult homozygous *BCAS3* knockout animals have been reported so far [17, 19]. The basic organization and structure of the brain in zebrafish and humans are highly similar, providing an excellent model for studying the molecular mechanisms of neurodevelopmental disorders [31, 32]. In this study, we constructed *bcas3* KO zebrafish to study the potential pathogenic mechanisms of *BCAS3* mutations. Morphology analysis and behavior tests revealed that *bcas3* KO zebrafish exhibit developmental defects and behavior impairments similar to those observed in patients with *BCAS3* mutations.

Deficiency of *BCAS3* in humans results in globally delayed development, while knockout of *Bcas3* in mice leads to premature death with aberrant cardiovascular patterning during early development and *rudhira*-RNAi Drosophila exhibited varying degrees of development abnormalities or death at different temperatures [12, 15, 16]. The *bcas3* KO zebrafish survived to adulthood but exhibited global developmental delay at early stages, making them more suitable for studying the long-term phenotypes of neurodevelopmental disorders. *Bcas3* KO in zebrafish caused microcephaly, reduced body length and impaired locomotor activity, consistent with the phenotypes observed in patients with *BCAS3* mutations, such as severe global developmental delay, microcephaly, and short stature [11, 12]. Additionally, *bcas3* KO in zebrafish increased locomotor activity and anxiety-like behavior during light-dark alternating stage, indicating impaired motor coordination. This may be due to abnormal development of motor centers in the brain, similar to the motor disorders observed in patients with *BCAS3* mutations.

We further explored the potential role of *bcas3* on behavior in adult fish by conducting a panel of behavior tests. Based on the results, *bcas3* KO zebrafish exhibited abnormal exploratory behavior with higher anxiety level observed in the novel tank test and increased aggressivity in mirror test, which are consistent with the behavioral phenotypes observed in patients with *BCAS3* mutations, such as motor disorder and hyperreflexia. These findings indicate *bcas3* KO leads to short-term memory dysfunction. In addition, the reduced social interaction in the shoaling test and social interaction behaviors tests further supports the role of *BCAS3* in regulating brain development. Taken together, the abnormal behaviors in *bcas3* KO zebrafish provide strong evidence that *bcas3* plays an important role in modulating multiple behaviors in vertebrates.

To shed light on the molecular pathways affected by defective *BCAS3*, we carried out transcriptome analysis using RNA-seq. The expression of genes related to startle response, embryonic brain development and neuron migration were significant altered. Among them, the expressions of *cyfip2* and *erbb3b*, which are associated with startle response were reduced in *bcas3* KO zebrafish. Haploinsufficiency of *Cyfip2* in mice caused developmental and epileptic encephalopathy, including microcephaly and abnormal social behaviors, such as increased anxiety [33–36]. *Erbb3b* knockdown lead to increased mortality and malformation due to a reduction in enteric neurons in zebrafish [37–39]. The expression level of *rpl10* involved in embryonic brain development is downregulated. Studies showed that loss-of -function variants in *RPL10* caused X-linked microcephaly and increased apoptosis in the brain [30, 40, 41]. Precise positioning of neurons is critical for their functional input and output, and the altered expression level of genes related to neuron migration may contribute to abnormal brain development [42]. Together with the increased apoptosis observed in the brain of *bcas3* KO zebrafish, these results demonstrate an important role for BCAS3 in brain development of zebrafish.

The increased apoptosis in the brain of *bcas3* KO zebrafish, marked by heightened AO staining and a decreased Bcl2/Bax expression ratio, likely impairs neuronal survival and brain development, contributing to the developmental delays and behavior abnormalities observed in *bcas3* KO zebrafish. Additionally, BCAS3 promotes p53 ubiquitination and proteasomal degradation through the CRL4A complex, reducing p53 stability [28]. In A172 cells, *BCAS3* knockdown significantly upregulated the expression of p53, thereby triggering the p53/GADD45α signaling pathway [27]. The accumulation of p53 results in increased apoptosis by disturbing the balance between the expression of Bcl2 and Bax [43]. This p53-mediated apoptosis may underlie the developmental abnormalities observed in *bcas3* KO zebrafish, including microcephaly due to the loss of neural cells or disruptions in neural network connectivity [44]. Additionally, the increased anxiety-like behaviors, aggressivity, and reduced social behaviors may reflect p53-induced apoptosis, impairing the function of neurons in motor centers [45], such as cerebellum or spinal cord. These findings highlight the critical role of the BCAS3-p53-Bax/Bcl2 axis in regulating apoptosis and its impact on brain development and behavior, providing a novel potential mechanistic link between *BCAS3* deficiency and neurodevelopmental disorders.

In conclusion, the *bcas3* KO zebrafish showed similar phenotypic characteristics to those observed in patients with *BCAS3* mutations, including delayed development, microcephaly, enhanced apoptosis and abnormal behavior. Thus, our work provides a robust and versatile model for studying the phenotypes and mechanisms of neurodevelopmental disorders caused by *BCAS3* mutations.

## Materials and methods

### Ethical statement

The husbandry and experimental procedures for zebrafish were approved by the Ethics Committee on Animal Research of Huazhong University of Science and Technology, adhering to the guidelines outlined in the National Institutes of Health Guide for the Care and Use of Laboratory Animals.

### Zebrafish maintenance

The zebrafish strains were maintained in standard laboratory conditions. Wild-type zebrafish of A.B. strain was obtained from the National Zebrafish Resource Center. Zebrafish embryos were maintained in embryo medium at 28.5 ℃.

### Generation of *bcas3* knockout zebrafish

Establishing zebrafish *bcas3*-deficient mutant line was performed by microinjecting recombinant Cas9 protein (INNOBIO, Ningbo) and *bcas3*-specific sgRNA. The guide sequence was designed using the online design tool CHOPCHOP. The target guide sequence for exon 12 of *bcas3-203* (ENSDART00000149957.3) was 5’- GGTCACAACCTTGCCCACCATGG-3’ and synthesized using TranscriptAid T7 High Transcription Kit (Thermo Scientific). Mutations in F0 fish (the treated gestating dam) were identified by Sanger DNA sequencing.

### Zebrafish genotyping

For determining the genotype of adult zebrafish, genomic DNA was extracted from tail fin of adult zebrafish. PCR amplification of Exon 12 in *bcas3* gene was performed with below primers.

Forward primer: 5’- GACACCACCTTAAACATACTG-3’;

Reverse primer: 5’- GATCATGATGTATAACGAGG-3’.

### Morphological evaluation of *bcas3* KO zebrafish

Zebrafish embryos were selected and photographed at different developmental stages. The interorbital distance and body length of *bcas3*^*−/−*^ zebrafish at 5 dpf were measured. Also, the body length and brain weight of 4 mpf zebrafish were measured. The zebrafish were treated with 0.01% MS-222 (Aladdin, #E017465) and lateral and dorsal images were captured using the Olympus SZX16 microscope (Olympus, Tokyo, Japan) equipped with an Olympus DP72 digital camera using cellSens software (version1.6; Olympus).

### Behavioral analysis

#### Spontaneous curls

28 hpf zebrafish embryos removed chorion were placed into embryo medium for 1 h. The number of embryo tailings in 20 s was recorded under stereo microscope.

#### Movement test

Zebrafish larvae were placed in 96-well plates at 28 ℃. After a 30-minute acclimation period at 28 ℃, the zebrafish larvae were recorded for 30 min using a DanioVision behavior chamber (Noldus Information Technology, Wageningen, the Netherlands).

#### Light-dark cycle test

6 dpf zebrafish larvae were transferred to 96-well plates individually. Following a 30-minute habituation period, the zebrafish larvae were observed and recorded for an additional 30 min followed by five light-dark transition cycles, each cycle consisting of 5 min of light followed by 5 min of darkness and 25 min light.

#### Novel tank test

The test utilized a transparent, rectangular tank (29 cm in length, 9 cm in width and 20 cm in height). The tank was divided horizontally into three equal sections: the top, middle, and bottom zones. WT and *bcas*3 KO zebrafish at 4 mpf, reared under identical conditions, were individually placed in the tank filled with water. Reduced time spent in the top zone of the novel tank indicates increased anxiety-like behavior [46]. Following a 5-minute acclimation period, the swimming behaviors of zebrafish were recorded for 6 min using a video camera positioned to capture a lateral view. The recorded videos were subsequently analyzed using Noldus tracking software (EthoVision XT v.14.0.1314) to quantify the movement distance and time spent in each zone.

#### Mirror test

The tank was measured 29 cm in length, 9 cm in width and 15 cm in height, with a mirror positioned on one side. According to the horizontal distance to the mirror, the tank was divided into three zones, including contact zone (< 2 cm from the mirror), approach zone (2–6 cm from the mirror) and far zone (> 6 cm from the mirror). Increased time spent in the contact zone indicates heightened aggressive behavior [47]. WT and *bcas3* KO zebrafish at 4 mpf, reared under identical conditions, were individually placed on the opposite side of the mirror. Following a 5-minute acclimation, the swimming behaviors of zebrafish were recorded for 6 min. The recorded videos were subsequently analyzed using Noldus tracking software (EthoVision XT v.14.0.1314) to quantify the movement distance and time spent in each zone.

#### Shoaling test

Six 4 mpf zebrafish of the same genotype were released in the center of the transparent tank (40 × 40 × 20 cm). After acclimating for 5 min, the behavior of zebrafish was recorded 5 min. The camera was fixed at the side views of the tank. The zebrafish-internal distances between the farthest two fish were estimated every 20 s using Adobe Photoshop to determine group cohesion and shoaling tendencies.

#### Social test

The test utilized a tank (20 × 10 × 10 cm) divided into two compartments by a transparent partition. A colored opaque divider was placed over the transparent partition to block the view of the test zebrafish. WT and *bcas3* KO zebrafish at 4 mpf, reared under the same conditions, were individually placed into the tank. Prior to testing, a stimulus group consisting of three 4 mpf zebrafish was placed in the right compartment. Fresh zebrafish were used as stimuli for each trial to ensure active behavior. After acclimating for 5 min, the colored divider was removed and the swimming behaviors of zebrafish was recorded. The recorded videos were subsequently analyzed using Noldus tracking software (EthoVision XT v.14.0.1314) to quantify the average velocity and time spent in each zone.

### RNA sequencing and bioinformatics analysis

Total RNA was extracted from whole body of wild-type and *bcas3* KO larvae at 3 dpf using Trizol reagent (Invitrogen), with 30 larvae per group. The quality of total RNA was quantified by 1% agarose gel, NanoDrop (Thermo Fisher Scientific Inc) and Agilent 2100 Bioanalyzer (Agilent Technologies). Next generation sequencing libraries were prepared according to the manufacturer’s protocol (NEBNext^®^ Ultra™ RNA Library Prep Kit for Illumina^®^). The libraries were sequenced on an Illumina HiSeq platform. After the transcriptome assembly, StringTie was utilized for estimating the expression levels of all transcripts, calculating mRNA expression levels as FPKM. The identification of differentially expressed mRNAs was performed using the R package DESeq2, with a selection criterion of |log2 fold change| ≥ 2 and P value < 0.01. GO-Term Finder was used to identify Gene Ontology (GO) terms enriched in a list of significant genes (p-value < 0.05). GESA (Gene Set Enrichment Analysis) was used to detect changes in expression of gene set enrichment analysis rather than individual genes.

### RNA isolation and quantitative real-time PCR

Total RNA was isolated from whole larvae at different time points or 4 mpf zebrafish brains of each genotype using Trizol reagent (Invitrogen). cDNA was synthesized from 1 µg of total RNA with HiScript^®^ II Q RT SuperMix according to the manufacturer’s protocol (Vazyme).

For RT-PCR, the following cycling conditions were used: 5 min at 95 °C, 25 cycles of 30 s at 95 °C, 30 s at 56 °C and 30 s at 72 °C, and a final step at 72 °C for five minutes. The PCR products were separated in 1% agarose gel by electrophoresis. Quantitative real-time RT-PCR analysis was performed using the AceQ qPCR SYBR Green Master Mix (Low ROX Premixed) on a QuantStudio 3 Flex Real-time PCR System (Applied Biosystems). The *actb* gene that encodes β-actin was used as an internal control. The sequences of primers used for real-time RT-PCR analysis are listed in Tabel S1.

### Western blot

 Zebrafish were harvested at 3 dpf and washed three times by PBS. The zebrafish were kept in a RIPA lysis buffer (Beyotime). Proteins were denatured by heat at 100 °C and separated by 8% NuPAGE and transferred to a nitrocellulose membrane and nonspecific binding was blocked with 5% nonfat dry milk in TBST for 2 h at room temperature. With constant shaking, membranes were incubated with primary antibodies overnight at 4 °C. Subsequently, the membranes were incubated with secondary antibodies for 2 h with constant shaking. Primary antibodies: Bcl2 (Zenbio, #381702), Bax (Abmart, #TA0120), β-Tubulin (Abmart, #M2005). Secondary antibodies used for Western blotting were horseradish peroxidase goat anti-mouse (CWBIO, #C0102S) and horseradish peroxidase goat anti-rabbit (CWBIO, #CW0103S).

### Whole-mount in situ hybridization

The collected zebrafish embryos were fixed with 4% paraformaldehyde (PFA) at 4℃ overnight. After 5 min washed twice with PBST, embryos were treated with Proteinase K/PBST. Subsequently, embryos were fixed with 4% PFA for 20 min at room temperate (RT), followed by 5-minute washed twice with PBST. RNA probe solution was then applied to embryos, which were sealed with coverslips and incubated in a humidified chamber at 65 °C for 14 to 16 h. Embryos were washed with 5×SSCT/5% formamide, followed by incubation with 2×SSCT/50% formamide at 65 °C for 1 h. Subsequent washes were performed with 2×SSCT at 65 °C for 15 min, followed 0.2×SSCT for 15 min and PBS for 5 min. After washing, embryos were blocked for 3 h at RT and then incubated with anti-dig (1:400) solution at 4 °C overnight. Embryos were washed with PBS three times (30 min/each wash) and with AP buffer for 5 min. Afterwards, embryos were transferred to 24 wells and incubated with NBT/BCIP (Sigma-Aldrich, #72091) (diluted in AP buffer, 1:200) for 1 h at RT. After three washes with PBST, embryos were fixed with 4% PFA/PBS at 4 °C overnight. Following three washes with PBST, embryos were imaged using the Olympus SZX16 microscope (Olympus, Tokyo, Japan) equipped with an Olympus DP72 digital camera and cellSens software (version1.6; Olympus). Primer sequences of *bcas3*:

Forward primer: 5’- TGTGGCGTTTTGTGAGGACCT-3’;

Reverse primer: 5’- TAATACGACTCACTATACGGCGGGTACCACTGTGAGCT-3’.

### AO staining

Embryos recovered at 36 hpf were incubated in 5 µg/mL AO stain (Sangon Biotech) dissolved in embryo medium in the dark for 30 min. After incubation larvae were washed 3 times for 5 min each embryo medium to remove residual dye. The fluorescent images were obtained with the Nikon fluorescence microscope.

### Construction of plasmid

The coding region of human *BCAS3* was PCR-amplified from MCF-7 cell lines cDNA, and fused into the pcs2-EGFP vector, resulting in pcs2-*BCAS3*. The pcs2-EGFP plasmid and pcs2-*BCAS3* plasmid was linearized with Not I restriction cut, and used for preparation of capped mRNAs using T7 RNA polymerase and the mMESSAGE Mmachine system (Ambion). The sequences of primers used for plasmid construction was listed in Tabel S2.

### Statistical analysis

All experiments were independently performed at least three times. The data were combined and analyzed together. Quantitative data were shown as means ± standard error of mean (SEM). An unpaired Student’s *t*-test and One-way ANOVA were used to compare the means from different groups, with significance *P < 0.05, **P < 0.01, ***P < 0.001. ns, not significant.

## Electronic Supplementary Material

Below is the link to the electronic supplementary material.

Supplementary Material 1

## Data Availability

All data are available in the main text or the supplementary materials.
