# Supplementary material for: Knockout of bcas3 gene causes neurodevelopment defects in zebrafish
Source: Biol Res. 2025 Jun 6;58:34. doi: 10.1186/s40659-025-00615-4 (PMC12142951; doi:10.1186/s40659-025-00615-4)
Supplement: Supplementary file 1 — Supplementary Material 1 [file 40659_2025_615_MOESM1_ESM.docx]

**SUPPLEMENTARY MATERIALS**

**Table S1. Primer for real-time RT-PCR**

| Gene | Sequence |
| --- | --- |
| *bcas3*-F | TGTGGGATCTGGAACAGCAG |
| *bcas3*-R | GGATGGTGGGAAATCGTGGA |
| *cyfip2*-F | CTTCAAGCTTCAGGTGGTGC |
| *cyfip2*-R | CACCATCTGCTCACACAGGT |
| *erbb3b*-F | CGGGCACTTTGTTGTGGATG |
| *erbb3b*-R | TAGGACACAGGCCAATGCAG |
| *eya4-*F | TGAGCCTCTGAACAGCAGTG |
| *eya4*-R | ATTGGCCTGTGTAGGCTGAC |
| *rpl10*-F | GTACGAGGCCGTTAAGGAGG |
| *rpl10*-R | ACGCAGACAGAGAACTTGGG |
| *nr2f1b*-F | TCGAGATACGGCAACCAGTG |
| *nr2f1b*-R | CGTTCAGCACGAACAACTCG |
| *prkg1b*-F | GGTGGATCTACACGGCGAAA |
| *prkg1b*-R | GCGTACTGGAGATCGCGTAA |
| *ackr3b*-F | AAGCATGAAGGAGTGGACGG |
| *ackr3b*-R | TCGACGTTCCTGGTCAACTG |
| *rpl13a*-F | GAGGAGCTGGAAGTTGGTGT |
| *rpl13a*-R | TCCGATAGTGCAGAGGCATA |

**Table S2. Primer for human BCAS3 plasmid**

| Gene | Sequence |
| --- | --- |
| *BCAS3*-F | GAATTCATGAATGAAGCTATGGCTACAGA |
| *BCAS3*-R | GGATCCGCTCAGAGTCTCGATGCTTCCCT |
